# A westernized diet changed the colonic bacterial composition and metabolite concentration in a dextran sulfate sodium pig model for ulcerative colitis

**DOI:** 10.3389/fmicb.2023.1018242

**Published:** 2023-04-17

**Authors:** Farhad M. Panah, Katrine D. Nielsen, Gavin L. Simpson, Anna Schönherz, Andreas Schramm, Charlotte Lauridsen, Tina S. Nielsen, Ole Højberg, Marlene Fredborg, Stig Purup, Nuria Canibe

**Affiliations:** ^1^Department of Veterinary and Animal Sciences, Aarhus University, Tjele, Denmark; ^2^Department of Biology, Aarhus University, Aarhus, Denmark

**Keywords:** inflammatory bowel disease, ulcerative colitis, meat consumption, colonic inflammation, porcine model, 16S rRNA gut metagenomics, dextran sulfate sodium

## Abstract

**Introduction:**

Ulcerative colitis (UC) is characterized by chronic inflammation in the colonic epithelium and has a blurred etiology. A western diet and microbial dysbiosis in the colon were reported to play a role in UC development. In this study, we investigated the effect of a westernized diet, i.e., increasing fat and protein content by including ground beef, on the colonic bacterial composition in a dextran sulfate sodium (DexSS) challenged pig study.

**Methods:**

The experiment was carried out in three complete blocks following a 2×2 factorial design including 24 six-week old pigs, fed either a standard diet (CT) or the standard diet substituted with 15% ground beef to simulate a typical westernized diet (WD). Colitis was induced in half of the pigs on each dietary treatment by oral administration of DexSS (DSS and WD+DSS, respectively). Samples from proximal and distal colon and feces were collected.

**Results and discussion:**

Bacterial alpha diversity was unaffected by experimental block, and sample type. In proximal colon, WD group had similar alpha diversity to CT group and the WD+DSS group showed the lowest alpha diversity compared to the other treatment groups. There was a significant interaction between western diet and DexSS for beta diversity, based on Bray-Curtis dissimilarly. The westernized diet and DexSS resulted in three and seven differentially abundant phyla, 21 and 65 species, respectively, mainly associated with the Firmicutes and Bacteroidota phyla followed by Spirochaetota, Desulfobacterota, and Proteobacteria. The concentration of short-chain fatty acids (SCFA) was lowest in the distal colon. Treatment had a slight effect on the estimates for microbial metabolites that might have valuable biological relevance for future studies. The concentration of putrescine in the colon and feces and that of total biogenic amines was highest in the WD+DSS group. We conclude that a westernized diet could be a potential risk factor and an exacerbating agent for UC by reducing the abundance of SCFA-producing bacteria, increasing the abundance of pathogens such as *Helicobacter trogontum*, and by increasing the concentration of microbial proteolytic-derived metabolites in the colon.

## 1. Introduction

Ulcerative colitis (UC) is a human inflammatory bowel disease (IBD) characterized by chronic, nonspecific inflammation in the colonic epithelium (Granlund et al., 2013) as a result of chronic dysregulation of the immune response in colonic mucosa (Eichele and Kharbanda, 2017). While the exact etiology of IBD is not yet known, host genetic predisposition (McGovern et al., 2015), aggravated immune response to host gut microbiota (Roselli and Finamore, 2020), and environmental factors such as diet and altered gut microbiota have been shown to play a role in the incidence of IBD (Guo et al., 2020; Galipeau et al., 2021). Consumption of a western diet, characterized by being rich in animal protein and fat and low in dietary fiber, is considered a main factor contributing to increased UC risk (Chiba et al., 2019). Red meat is one of the ingredients of a typical western diet (Thøgersen and Bertram, 2021). In human studies, red meat consumption has been associated with UC, by inducing or exacerbating this disease (Jantchou et al., 2010; Ge et al., 2015; Rashvand et al., 2015) due to increased proteolytic activity by the colonic bacteria resulting from higher substrate availability (Gilbert et al., 2018). Although the exact mechanism behind this effect is yet to be elucidated (Van Hecke et al., 2021), microbial fermentation of undigested dietary and endogenous protein sources results in various metabolites, several of which, e.g., biogenic amines, NH_4_^+^, indoles, phenols, and H_2_S, are considered detrimental for gut health as they are capable of stimulating the immune system and starting a cascade of inflammatory responses in the colon (Windey et al., 2012; Gilbert et al., 2018; Panah et al., 2021). These metabolites can result in the loss of microvilli and apoptosis of colonocytes, which are ultrastructural changes in the colon at the early stages of diarrhea due to colitis (Thomson, 2009; Gilbert et al., 2018). Red meat intake leads to the formation of reactive oxygen species and it may also damage the colonic epithelium through increased concentrations of heme (originated from red meat haemoglobin), and amines (Glei et al., 2006). Accordingly, Le Leu et al. (2013) reported that feeding red meat to mice with colitis (induced by dextran sulfate sodium; DexSS) exacerbated colitis as observed by increased histopathological scores in the colon and changes in the gut microbial composition.

Dysbiosis of colonic microbiota was reported to be associated with IBD (Ni et al., 2017) as gut microbiota are involved in various physiological functions, critical in colonic inflammation such as energy and nutrient supply (Ng et al., 2013), maintenance of mucosal homeostasis (Zheng et al., 2020), and modulation and enhancement of host immunity (Yang et al., 2021). Dysbiosis has been defined as reduced biodiversity, abnormal and varying composition of large intestine microbiota, alongside changes in the interactions among different strains of microbiota and between microbiota and the host (Guo et al., 2020). However, since mucosal inflammation in the colon can lead to dysbiosis, it remains unclear whether microbial dysbiosis is the cause or the consequence of IBD (Ni et al., 2017). It has been speculated that increased fecal proteolytic activity, associated with changes in gut microbiota, preceding the clinical diagnosis of UC, is potentially indicative of a causal relationship (Galipeau et al., 2021).

Studies have shown that patients with UC may have a less diverse bacterial community in the large intestine, with lower abundance of Firmicutes, Bacteroidetes and Verrucomicrobia phyla, and higher abundance of Proteobacteria (Manichanh et al., 2012; Zakerska-Banaszak et al., 2021). Microbial dysbiosis, in return, may alter metabolic conditions in the gut (Yang et al., 2021) by reducing carbohydrate-fermenting bacteria such as Lachnospiraceae (in particular *Roseburia* spp.), and Prevotella and Ruminococcus taxa, which are involved in production of short-chain fatty acids (SCFAs; Louis et al., 2007). Short-chain fatty acids such as acetate, butyrate and propionate, and lactate are produced from bacterial fermentation of indigestible carbohydrates, and to a lesser extent from protein, in the colon, and exert vital beneficial effects to the maintenance of mutualistic relationships between commensal bacteria and the host immune system (Rooks and Garrett, 2016). The intestinal immune system is affected by SCFAs, since they inhibit pathogenic growth (Vinolo et al., 2011), regulate inflammatory responses in the colon (Maslowski et al., 2009), participate in secretion of the epithelial repair cytokine interleukin-18, and they also enhance the integrity of epithelial cells by promoting goblet cell mucin production and modification of tight junctions (Guo et al., 2020). Among SCFAs, butyrate has a significant biological importance in the context of IBD. It is the preferred energy source for colonocytes (supplying 70–80% of their required energy; Galipeau et al., 2021; Gasaly et al., 2021), it regulates immune responses by activation of G protein-coupled receptors (GPRs; Sanchez et al., 2020), it renders the differentiation of monocytes to macrophages through inhibition of histone deacetylase (HDAC; Schulthess et al., 2019), and its oxidation by colonocytes generates hypoxia, helping to maintain luminal anaerobiosis, which is favorable for the hindgut microbiota (Gasaly et al., 2021). In a number of studies, IBD patients exhibit lower abundance of butyrate-producing bacteria, lower butyrate concentration (Marchesi et al., 2007) and depressed colonocyte butyrate oxidation, which lowers luminal anaerobiosis and facilitates the expansion of bacteria like *Enterobacteriaceae* that contribute to inflammation (Gasaly et al., 2021). Vieira et al. (2012) showed that oral administration of butyrate to mice with UC induced by dextran sulphate sodium (DexSS), ameliorated the inflammatory profile of the colon mucosa, possibly by activation of GPR109A, which contributes to the suppression of DexSS-induced acute colitis (Yang et al., 2021).

Aimed at demystifying the complex pathology of IBD, many studies have been conducted using animal models (Knudsen et al., 2020; Mizoguchi et al., 2020; Nielsen et al., 2020; Mizoguchi et al., 2021). Colitis in pigs may share some symptoms with UC (Chase-Topping et al., 2007; Panah et al., 2021), and since pigs are closer to humans than rodents like mice or guinea pigs with regard to their colonic microbiota, anatomy and physiology (Liu et al., 2017), they are often used as surrogate models. Therefore, establishing inflammation in the colon of pigs could provide a better experimental model for human IBD (Pistol et al., 2020). One of the most common chemicals used to simulate active IBD in animal models is DexSS administered orally, which has previously been tested in pig (Nielsen et al., 2020; Pistol et al., 2020) and murine (Eichele and Kharbanda, 2017) colitis models. It has been combined with dietary interventions to investigate, e.g., the potential impact of food components on mitigation of IBD activity (Bassaganya-Riera and Hontecillas, 2006; Pistol et al., 2020).

We have previously shown that offering a westernized diet to 6-week old pigs by replacing 15% of the pelleted standard diet with cooked minced beef 2 weeks prior to DexSS treatment, aggravated the severity of colitis (Nielsen et al., 2020). Using samples from these piglets in the current study, we aimed at investigating the impact of feeding a westernized diet on colonic and fecal microbial composition and microbial metabolites. We hypothesized that the exacerbating effect of a westernized diet on colitis in DexSS-challenged pigs is associated with changes in the gut microbiota induced by the diet composition.

## 2. Materials and methods

### 2.1. Ethics statement

The care and housing of the animals used in the experiment complied with Danish laws and regulations for the humane care and use of animals in research (The Danish Ministry of Justice, Animal Testing Act no. 1306 of 23 November 2007) and was performed under the license obtained from the Danish Animal Experimentation Inspectorate, Ministry of Food, Agriculture and Fisheries.

### 2.2. Experimental design

A total of 24 pigs in three complete blocks following a 2 × 2 factorial design was used, as described by Nielsen et al. (2020). Briefly, 24 6-week old pigs were fed either a standard weaner diet (Prime Midi Piller U; DLG, Randers, Denmark) or a diet in which 15% of the standard diet (by weight of the total pelleted diet) was substituted with minced, cooked and dried ground beef (Hørkram Foodservice, Hørning, Denmark) to simulate a westernized diet from experimental day 0 to 14. Throughout the three blocks of the experiment, two non-littermate pigs from different sows were housed in one pen to receive the same treatment, and piglets from two different sows were used in each block. The animals were fed one of the two experimental diets for 14 days. From day 14, an oral dose of DexSS was administered to half of the pigs fed the standard diet (DSS; *n* = 5) and half of the pigs fed the WD diet (WD + DSS; *n* = 6); the other half continued without DexSS administration to the end of the experiment, forming the control (CT; *n* = 6) and westernized diet (WD; *n* = 6) groups, respectively. One of the pigs treated with DexSS reached the humane end point prior to the end of experiment, hence it was euthanized and results are not included in the downstream analysis; therefore, the results are from total number of 23 pigs. The DexSS treatment was administered as 1.25 g DexSS (MW 36–50 kDa, MP Biomedicals, Santa Ana, CA, United States)/kg BW, dissolved in 20 ml of sterile saline solution (0.9% NaCl) + 5 ml apple juice. On days 18 and 19, fecal samples were collected (1.5 g) and, on day 19, all animals were sacrificed and colonic digesta were collected from the proximal (25% of colon length) and distal colon (75% of colon length) for subsequent metabolite (4.0 g) analysis and DNA extraction (1.5 g). Fecal and digesta samples for DNA extraction were snap frozen in liquid nitrogen and stored at −80°C until analysis. Samples for metabolite analysis were stored at −20°C.

### 2.3. DNA extraction and 16S rRNA gene sequencing

Total DNA was extracted from 200 mg fecal (collected on day 18) and digesta (collected on day 19) samples using the E.Z.N.A. stool DNA Kit (Omega bio-tek) according to the manufacturer’s instructions. Amplicon libraries of the bacterial 16S rRNA gene were prepared according to Illumina’s 16S Metagenomic Sequencing Library Preparation protocol (Illumina, 2013), with few modifications as described in Tawakoli et al. (2017). Briefly, 20 PCR cycles were performed with the bacterial primers Bac341F and Bac805R (Herlemann et al., 2011) to applify V3-V4 regions of 16S rRNA gene, followed by 10 cycles to add the Illumina adapters and 8 cycles to add the sequencing barcodes. The pooled libraries were sequenced on a MiSeq desktop sequencer (Illumina) using 2 × 300 bp chemistry (Illumina) according to the manufacturer’s instructions.

### 2.4. Bioinformatics analysis of 16S rRNA gene data

Raw sequencing reads were processed with Qiime 2 (Bolyen et al., 2019) using the DADA2 package (Callahan et al., 2016) for primer trimming, quality filtration, denoising, merging, chimera removal and inference of amplicon sequence variants (ASV). Forward and reverse primers were removed by truncating the forward (trim left forward: 17 nt) and reverse (trim left reverse: 21 nt) primer length from the raw sequence reads. Trimmed reads were quality filtered by truncating sequence reads at the points where 25% of reads dropped below a Phred quality score (Q) of 30 (truncation length of forward reads: 260; truncation length of reverse reads: 220). In this way, merging of the sequences took place with the minimum of 16 nucleotides overlap.

For diversity analysis, a phylogenetic tree was inferred using the fragment insertion method based on SATé-Enabled Phylogenetic Placement (SEPP) implemented in Qiime 2, which is inserting query sequences into an existing phylogenetic tree and aligning them to full-length sequences for the same gene (Mirarab et al., 2011).

For taxonomic classifications of detected ASVs, a region-specific classifier based on our primer set was created based on the SILVA v.138 (Quast et al., 2012) taxonomic reference database with 99% of similarity cutoff using the RESCRIPt plugin (Robeson et al., 2020) in Qiime 2. The classifier was trained using the Naïve-Bayes method and the output artifact was used to generate the classified taxonomy table. Our region-specific trained classifier is available on this directory: https://drive.google.com/file/d/1qwGbPvxhXJIC_bSmoB33Ala26LeN69BJ/view?usp=sharing.

All files generated in Qiime 2, i.e., ASV tables, ASV sequences, taxonomy table, and rooted phylogenetic tree were transferred to R by qiime2R package (Bisanz, 2018) for subsequent use with the phyloseq package (Holmes and McMurdie, 2013). ASVs belonging to taxonomic domain other than bacteria (e.g., Archaea and Eukaryotes) were removed and subsequent results are based only on bacteria. Decontamination of the reads was performed based on prevalence of ASVs in negative samples (*n* = 5) using the Decontam package (Davis et al., 2018) in R from which *Caldalkalibacillus uzonensis*, belonging to the phylum Firmicutes was identified as contaminant and removed from the ASV table. After this step, the negative control samples were deleted from the entire dataset. Additionally, ASVs with the prevalence in less than three samples (out of 66 samples) were filtered out. This resulted in filtering ASVs appeared in less than 5% of all samples. Moreover, singletons were removed from the dataset based on their abundance as described in this repository: https://github.com/farhadm1990/Microbiota-analysis (Panah, 2022). Singletons were those ASV that occurred only in one sample across all the dataset, i.e., their mean abundance was equal to their sum of abundance. Relative abundance of taxa was determined through dividing the number of sequencing reads assigned to different taxa in each sample by the total number of sequencing reads. Finally, all samples were normalized to the same reading depth of 17,000 reads per sample by rarefaction (sampling without replacement) in phyloseq. A total of 66 samples and 2,925 ASVs passed preprocessing and were used for the downstream analysis.

#### 2.4.1. Quantitative PCR and calculation of absolute abundance

Bacterial 16S rRNA gene copy numbers per g of sample were quantified in triplicate by quantitative PCR (qPCR) according to Busck et al. (2022). In brief, a 167 bp long gene fragment was amplified using the primer set Bac908F/Bac1075R (Mateos-Rivera et al., 2018), with annealing at 59°C and extension at 78°C. In order to obtain absolute abundances, relative taxon abundances derived from the sequencing reads were multiplied to the total load of bacterial 16S rRNA gene copies (total load per gram of sample), detected by qPCR (Jian et al., 2020). All results presented in this study are based on absolute count data.

#### 2.4.2. Alpha and beta diversity

Alpha diversity, a measure of microbiota diversity within a sample, was estimated based on richness or total number of observed ASVs (Chao1) and evenness using the Shannon’s index and on phylogenic distances using the Faith Phylogenetic Diversity (FaithPD) index. Chao1 and Shannon indices were estimated from the ASV count table using the phyloseq package; for estimation of FaithPD, the ASV count table and the rooted phylogenetic tree were used as the inputs in the *pd* function of picante package (Kembel et al., 2010). For Chao1, the non-rarefied ASV count table was used and for Shannon and FaithPD the rarefied ASV count table was used as the input.

Beta diversity, the difference in microbial composition between the treatment groups, was estimated by Bray–Curtis dissimilarity coefficients from the variance-stabilizing transformed (VST) ASV count table. Variance-stabilizing transformation was done using the *varianceStabilizingTransformation* function in DESeq2 package (Love et al., 2014).

#### 2.4.3. Analysis of differentially abundant taxa

Absolute ASV abundances were agglomerated to phylum and species level using the *tax_glom* function in phyloseq and all ASVs at family level classified as “uncultured” were removed. Normalization of the microbial data and differential abundance analysis was done using DESeq2 (Love et al., 2014) in R via a Negative Binomial Wald test statistics and associated *p*-value with the main effects of WD, DSS and their interaction as well as the effect of blocks and sample type as additional covariates. Before estimation of the dispersions, the geometric means of the counts in each sample were calculated and used to estimate size factors per sample using DESeq2 (Love et al., 2014). To correct for multiple hypothesis testing, *p*-values were adjusted for the False Discovery Rate (FDR) using the Benjamini-Hochberg (BH) method (Benjamini and Hochberg, 1995). ASVs were considered differentially abundant when the adjusted *p*-value ≤0.05 (FDR < 0.05) and a Log_2_ Fold Change (LFC) exceeding 0 (|LFC| > 0) at phylum level and |LFC| > 2 at species level.

### 2.5. Analysis of microbial metabolites

For quantification of chemical concentrations, fecal and digesta samples were collected in stomacher bags and kept on ice and stored in −20°C until the day of analysis. Concentration of the SCFAs acetate, propionate, butyrate, iso-acids (iso-butyrate and iso-valerate), and valerate were analyzed by capillary gas chromatography as described by Jensen et al. (1995) with some modifications as described by Canibe et al. (2007). Biogenic amines (cadaverine, agmatine, putrescine and tyramine) were quantified by gradient elution on reverse phase HPLC chromatography, as described by Poulsen et al. (2018). A heatmap of associations between bacterial absolute abundance and the concentration of different metabolites produced by colonic bacteria was created using the Spearman’s rank correlation method with the pheatmap package in R (Kolde and Maintainer, 2018). A significance test was performed to identify the significant associations and the pairwise comparisons were corrected for FDR < 0.05.

### 2.6. Statistical analysis

The relationships between predictor variables and the expected responses for alpha diversity metrics (i.e., Chao1, Shannon and FaithPD) and microbial metabolites were assessed using the R statistical software (Team R. C, 2021). A Generalized Linear Mixed-Effect Model with Gamma distribution and log link function was estimated using the *glmer* function of the *lme4* package (Bates et al., 2015). Estimated marginal means (EMM) of treatment effects were computed using the *emmeans* package (Lenth et al., 2018). The model estimated had the following functional form:


$$\begin{array}{c} \log\;\left(E\left(Y_{ijklm}\right)\right)=\alpha +W_{i}+D_{j}+W_{i}\cdot D_{j}+S_{k}+W_{i}\cdot S_{k}\\ +D_{j}\cdot S_{k}+W_{i}\cdot D_{j}\cdot S_{k}+B_{l}+P_{m} \end{array}$$

Where *Y* is the dependent variable and *α* the model constant term. The model includes the fixed effects of diet (*W_i_*; *i* = noWD and yesWD), DexSS (*D_j_*; *j* = noDexSS and yesDexSS), sample type (*S_k_*; *k* = digesta from proximal and distal colon, and feces), all second- and third-order interaction between these fixed effects, experimental block (*B_l_*; *l* = 1, 2, 3), and the random effect of pig (*P_m_*; *m* = 1, …, 23). We report EMMs with 95% confidence interval and pairwise comparisons between treatment groups from the full model. Pairwise comparisons were adjusted for false discovery ratio by BH method and declared significantly different where *p.adjust* ≤ 0.05.

Compositional differences between treatment groups were assessed using a distance-based redundancy analysis (dbRDA) based on Bray–Curtis dissimilarity matrix (litter effect used as a partial effect, whose effect was removed prior to estimation of treatment effects) in R using the vegan package (Oksanen et al., 2020) with 999 permutations (restricted by litter as a “block”-level restriction and by individual animal as plot strata, while permutation between segments was freely done; Simpson, 2022). Bray–Curtis dissimilarity was obtained from the *distance* function in phyloseq and the distance between samples in each treatment group was analyzed using non-metric multidimensional scaling (NMDS) using the *metaMDS* function in vegan (Oksanen et al., 2020) and phyloseq packages. The effect of segment was evaluated by a dbRDA model and tested with a permutation with restricted litter and samples from different sample type and free permutation between animals. The Bray–Curtis dissimilarity matrix was computed from log-transformed absolute counts. A test of homogeneity of group variances (dispersions around the centroids) of the four treatment groups was conducted prior to dbRDA with 999 restricted permutations (litter as a “block”-level restriction, animals were restricted via plot strata and observations from different segments were freely permuted) using the *betadisper* function in vegan. Unless otherwise stated, all multiple comparisons were corrected for FDR by the BH method and were declared significant at FDR ≤ 0.05.

The scripts for bioinformatics and statistical analysis are available at https://github.com/farhadm1990/Microbiota-analysis (Panah, 2022).

## 3. Results

### 3.1. Feed composition

Meat substitution of 15% by weight, resulted in increased protein and fat content of the diet by 40.8 and 66.7%, respectively, and reduced fiber content by 16% in westernized diet compared to the standard diet (Table 1).

**Table 1:** Nutrient composition (% as-is basis) of the standard and westernized diet (WD).

Item	Standard weaner diet^1^	WD^2^
Crude protein	19.1	26.9
Crude fat	4.8	8.0
Crude fiber	2.5	2.1
Crude ash	4.7	5.2
% Protein from meat	0	39.7
% Fat from meat	0	48.9
% Ash from meat	0	23.8

1 Nutrient composition in the standard diet was based on the declared composition by the feed company (DLG, Fredericia, Denmark).

2 Crude protein in ground beef was determined as *N* × 6.25 and N was measured by Dumas (Hansen, 1989). Concentration of crude fat in ground beef was quantified using the Stoldt procedure (Stoldt, 1952). Total ash was analyzed according to the AOAC method (923.03; AOAC, 1990).

### 3.2. Alpha diversity in digesta and feces

Average alpha diversity indices of feces and colonic samples from the experimental treatment are summarized in Table 2. There were no differences in alpha diversity between experimental blocks and sample type, i.e., proximal colon, distal colon, and fecal samples (Supplementary Table S1). In proximal and distal colon, WD group showed similar alpha diversity to CT group (Table 2). In proximal colon, the WD + DSS group had lowest Chao1, Shannon and FaithPD indices, while in distal colon, DSS group showed lowest alpha diversity (*p* < 0.05; Table 2). Chao1 and Shannon indices in distal colon were similar to that in DSS group. However, in fecal samples only FaithPD seemed to be affected by treatments and it was lowest in DSS group compared with WD group.

**Table 2:** Alpha diversity metrics with their estimated marginal means and their 95% confidence interval for each treatment group.

	Groups^1^			
	CT	WD	DSS	WD + DSS
Proximal
Chao1	533 (426–665)^b^	500 (399–625)^ab^	460 (360–588)^ab^	367 (296–462)^a^
Shannon	5.30 (5.0–5.66)^b^	5.20 (4.92–5.57)^b^	5.0 (4.63–5.30)^ab^	4.80 (4.05–5.09)^a^
Faith PD	35.4 (30.0–42.0)^b^	35.5 (30.0–42.0)^b^	29.4 (24.0–35.0)^ab^	27.4 (23.0–32.0)^a^
Distal
Chao1	535 (428–668)^b^	555 (443–695)^b^	397 (311–508)^a^	397 (315–501)^a^
Shannon	5.30 (4.94–5.59)^b^	5.30 (4.96–5.63)^b^	4.80 (4.46–5.12)^a^	4.90 (4.59–5.21)^ab^
Faith PD	36.4 (31.0–43.0)^b^	38.6 (32.0–46.0)^b^	26.9 (22.0–32.0)^a^	28.5 (24.0–34.0)^a^
Feces
Chao1	488 (387–615)	534 (426–668)	391 (301–506)	434 (347–542)
Shannon	5.20 (4.90–5.57)	5.30 (4.95–5.62)	5.0 (4.64–5.35)	5.0 (4.71–5.33)
Faith PD	34.3 (29–41)^ab^	37.9 (32–45)^b^	27.7 (23–34)^a^	30.6 (26–36)^ab^

1 Treatment groups: control (CT; *n* = 17), WD (CT + ground beef; *n* = 18), CT + dextran sulfate sodium (DSS; *n* = 14), WD + dextran sulfate sodium (WD + DSS; *n* = 17). Pairwise comparison for differences in EMMS between groups was adjusted with BH and EMMs are superscripted with different letters at *p* < 0.05.

### 3.3. Bacterial beta diversity of digesta and feces

**Figure 1:**
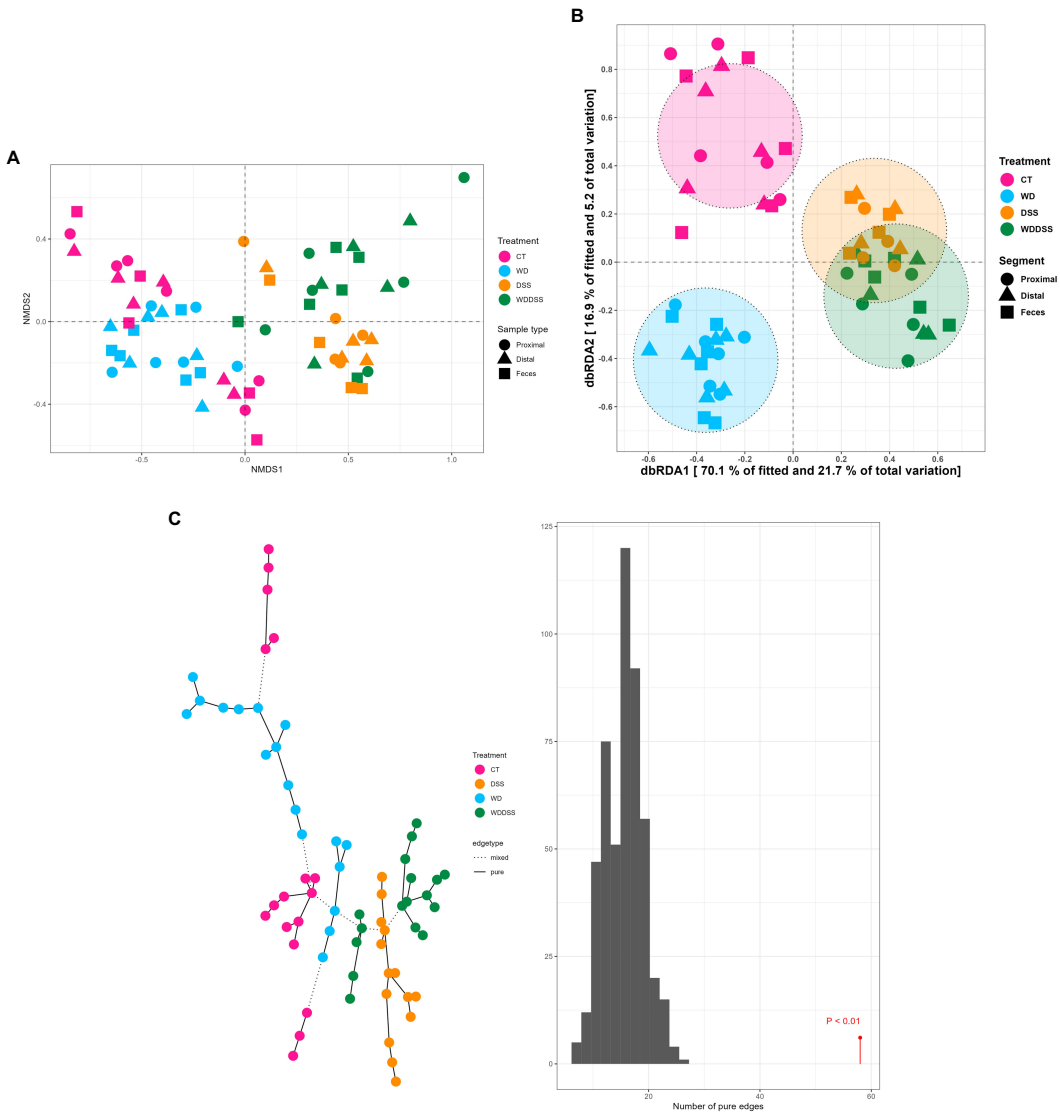
Non-metric multidimensional scaling (NMDS) plot based on Bray–Curtis dissimilarity index **(A)**, and dbRDA MDS plot **(B)** of sample scores from Bray–Curtis dissimilarity for different treatments (CT: control, WD: westernized diet, DSS: group treated with dextran sulfate sodium, and WDDSS: WD + DSS) extracted from dbRDA model (variance explained by our treatments = 31.0%, *p* < 0.01). On dbRDA plot, the treatments (constrained factors) explained 70.1 and 16.9% of the fitted variance and 21.7 and 5.20% of total variance for Bray–Curtis dissimilarity on dbRDA1 and dbRDA2 axis, respectively. The shape of the points represents the origin of the samples, i.e., digesta from proximal and distal colon, and fecal samples and the color of the points represent different treatments. Graph-based analysis of the distributions in bacterial composition for different treatments **(C)**, based on Bray–Curtis dissimilarity matrix with maximum distance of 0.35. The histogram of permutation test based on MST for Bray-Curtis is presented (*p* < 0.01).

The test for differences of bacterial diversity between the samples was done by dbRDA based on Bray–Curtis dissimilarity metric. To verify the validity of the test, the homogeneity of variance around the centroids for each treatment was examined (Supplementary Figure S1). Principal Coordinate Analysis (PCoA) plot shows the within group dispersion based on Bray-Curtis dissimilarities (Supplementary Figure S1) and illustrates the homogeneity of variances test (*betadisper*). This test showed no significant differences between groups in terms of dispersion around the treatment centroids (*F* = 5.12; *p* = 0.18). Accordingly, PCo1 and PCo2 explained 24.0 and 12.0% of the total variance, respectively. The results of the dbRDA model (Table 3) showed that there was a significant interaction between diet and DexSS (W·D; Pseudo-*F* = 10.8; *p* < 0.01) based on Bray-Curtis dissimilarities. However, the main effects of diet (Pseudo-*F* = 4.90; *p* < 0.01) and DexSS (Pseudo-*F* = 21.1; *p* < 0.01) were also significant. The magnitude of these effects was observed in samples from DexSS-treated groups based on the dbRDA model, showing that DexSS treatment explained 22.0% of the total variance (*p* < 0.01), while diet accounted for 5.0% of the total variance (*p* < 0.01) in the beta diversity based on Bray-Curtis dissimilarities. There was a distinct separation in Bray-Curtis dissimilarities on NMDS plot (Figure 1A) between treatment groups and the resolution of this separation became sharper when the sample scores of different treatments, extracted from dbRDA model, were plotted on PCoA plot (Figure 1B). According to the dbRDA plot of the sample scores, dbRDA1 explained 70.1% of the fitted variance and 21.7% of the total variance and dbRDA2 explained 16.9 and 5.20% of the fitted and the total variance, respectively. Although statistically significant, the effect of sample type on Bray–Curtis dissimilarity was small as it only explained 1.0% of the total variance based on the dbRDA model (Pseudo-*F* = 0.55; *p* < 0.01). Graph-based analysis on the Bray–Curtis dissimilarity matrix and permutation test based on Minimum Spanning Tree (MST) showed pure edges for each of the treatment groups, i.e., pure edges indicate similar bacterial composition, while mixed edge connections could also be seen between CT and WD, WD + DSS and DSS groups, and between WD + DSS and WD groups, i.e., mixed edges indicate samples with tendency to similar composition of bacteria (Figure 1C).

**Table 3:** Test statistics of the dbRDA model for the effect of treatment and sample type on Bray–Curtis dissimilarity.

	Degree of freedom	% of variance explained	Sum of squares	Pseudo-*F*	*p*-value
WD	1.0	5.0	0.05	4.90	0.02
DSS	1.0	22.0	0.23	21.1	<0.01
WD · DSS	3.0	31.0	0.33	10.8	<0.01
Sample type	2.0	1.0	0.12	0.55	<0.01

The values are averaged over three blocks.

### 3.4. Bacterial composition in digesta and feces

**Figure 2:**
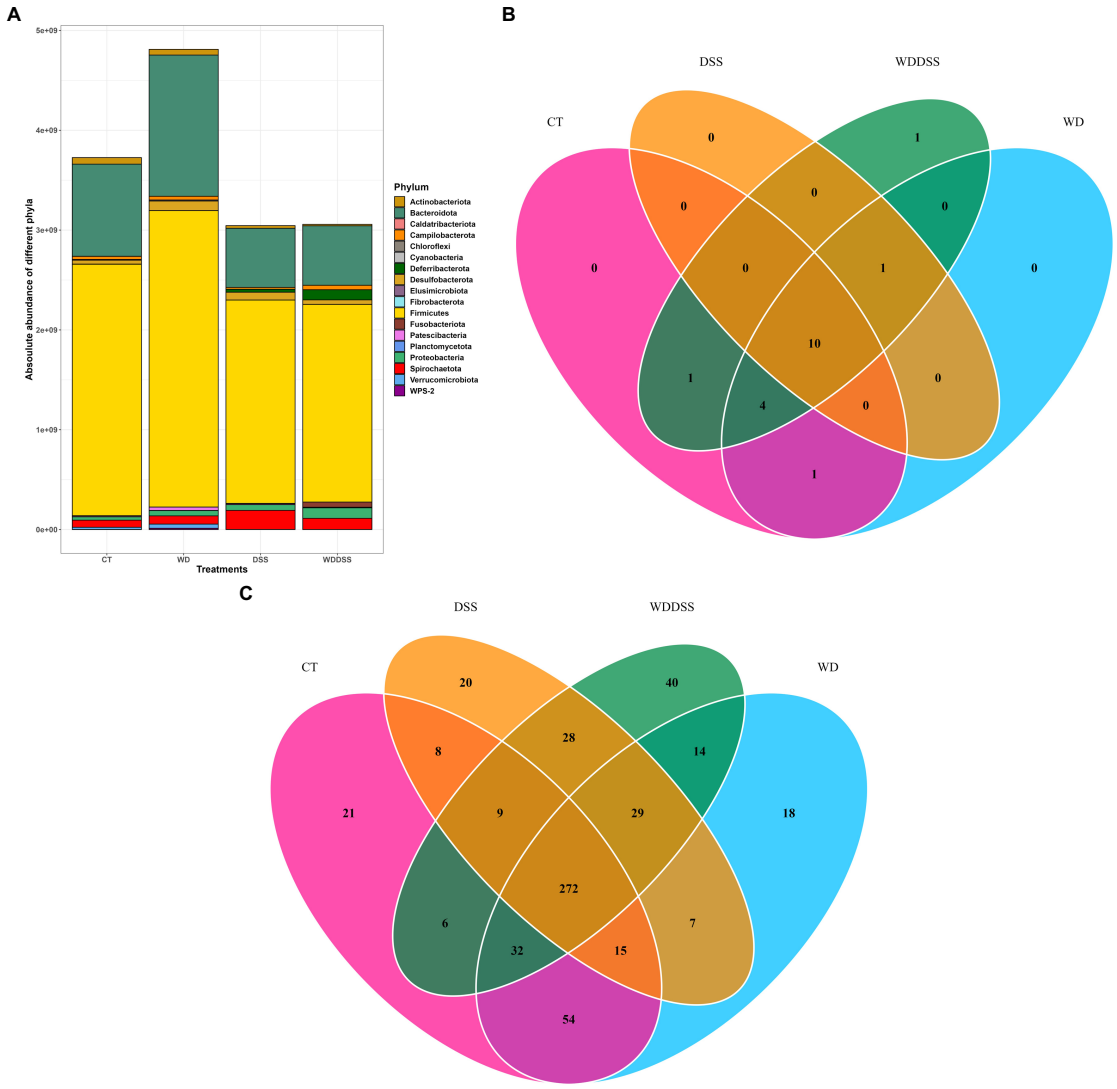
Absolute abundances of different phyla with total abundance across all sequence reads in different treatments **(A)**. The number of different phyla present in the treatment groups was 16, 16, 11, and 17 for CT, WD, DSS, and WDDSS (WD + DSS), respectively. Venn diagram of shared taxa between different treatments at Phylum **(B)** and Species **(C)** levels.

Our results showed that by targeting V3 and V4 of 16S rRNA gene, 175 out of 573 ASV (30.5%) was classified as known species and at genus level, 221 out of 235 unique genera (94%) were classified as known. Bacterial 16S rRNA gene load in the samples from feces, proximal and distal colon was analyzed by qPCR. Total bacterial load was 1.47 × 10^10^ 16S rRNA gene copies per g sample and it was slightly higher in WD group and lowest in DSS and WD + DSS groups. Regardless of the treatment, Firmicutes and Bacteroidota were the most abundant phyla in all groups, followed by Spirochaetota and Proteobacteria. The absolute abundance of Firmicutes was numerically lowest in WD + DSS and DSS compared to the CT and WD groups, 1.98 × 10^9^ and 2.04 × 10^9^ vs. 2.52 × 10^9^ and 2.97 × 10^9^ gene copies per g, respectively (Figure 2A). Absolute abundance of Bacteroidota and Fibrobacterota was lowest in the DSS and WD + DSS groups, and that of Spirochaetota was highest in the DSS and WD + DSS groups. Fusobacteriota was absent only in samples from the CT group, and Cyanobacteria and WPS-2 were absent in the DSS group (Figure 2A).

At Phylum level, 10 phyla were shared by all treatments: Actinobacteriota, Bacteroidota, Campilobacterota, Deferribacterota, Desulfobacterota, Firmicutes, Patescibacteria, Proteobacteria, Spirochaetota, and Verrucumicrobiota (Figure 2B). At species level, all groups shared 272 species and the most closely related groups, regarding uniquely shared species, were CT and WD, with 54 species in common followed by DSS and WD + DSS, with 28 shared species (Figure 2C).

### 3.5. Differential abundance analysis of taxa in feces and digesta

**Figure 3:**
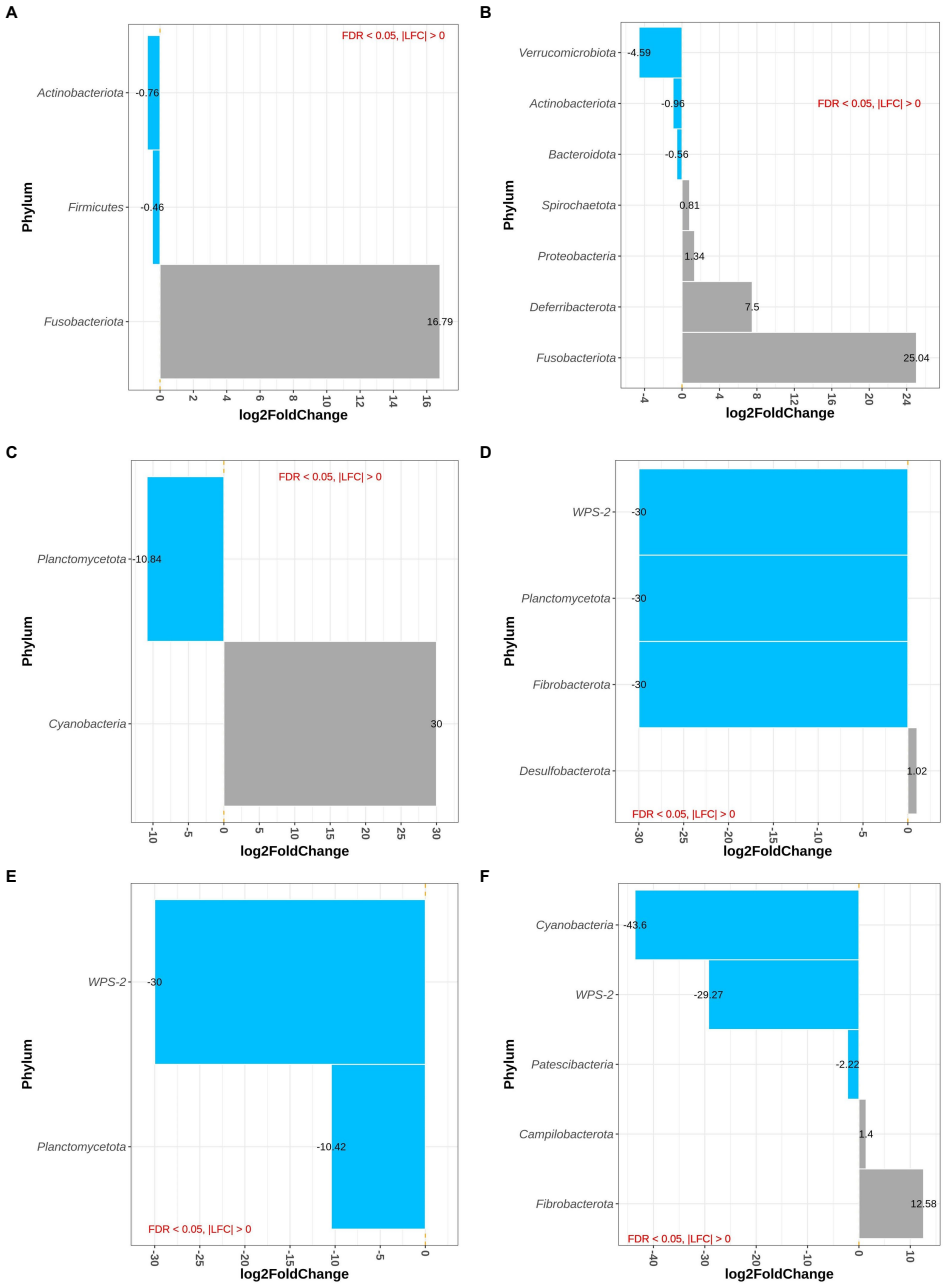
Differentially abundant Phyla for the main effect of diet (westernized diet groups vs. non-westernized group; **A**) and DexSS (DexSS groups vs. non-DexSS groups; **B**), and pairwise comparison for phyla with significant interaction for WD vs. CT **(C)**, DSS vs. CT **(D)**, WDDSS (WD + DSS) vs. CT **(E)**, and WDDSS (WD + DSS) vs. WD **(F)** with FDR < 0.05.

Sample type, i.e., proximal and distal colon and feces, had no effect on differential abundance of bacteria at phylum level. At phylum level, the main effect of westernized diet and DexSS resulted in 3 and 7 differentially abundant phyla, respectively (Figures 3A,B). Figure 3A shows that the animals fed with the westernized diet had low abundance of Actinobacteriota (LFC = −0.76) and Firmicutes (LFC = −0.46) and increased abundance of Fusobacteriota (LFC = 16.8). Administering DexSS resulted in a significant decrease in the abundance of Verrucumicrobiota (LFC = −4.59), Actinobacteriota (LFC = −0.96), and Bacteroidota (LFC = −0.56); and it increased the LFC for Spirochaetota (0.81), Proteobacteria (1.34), Deferribacterota (7.50) and Fusobacteriota (25.0). There was a significant interaction between diet and DexSS administration for differential abundance of 7 phyla, and pairwise comparisons of the different treatment groups for these phyla are presented in Figures 3C–F. In the WD, DSS and WD + DSS treatment groups, the abundance of Planctomycetota was reduced compared to the CT group. The WD group had higher abundance of Cyanobacteria with LFC = 30.0 (Figure 3C), and DSS (Figure 3D) and WD + DSS (Figure 3E) showed lower abundance of WPS-2 compared to the CT group. Comparing WD + DSS with WD, WD + DSS had higher abundance of Fibrobacterota and Campilobacterota, while it showed lower abundance of Cyanobacteria and WPS-2 (Figure 3F).

**Figure 4:**
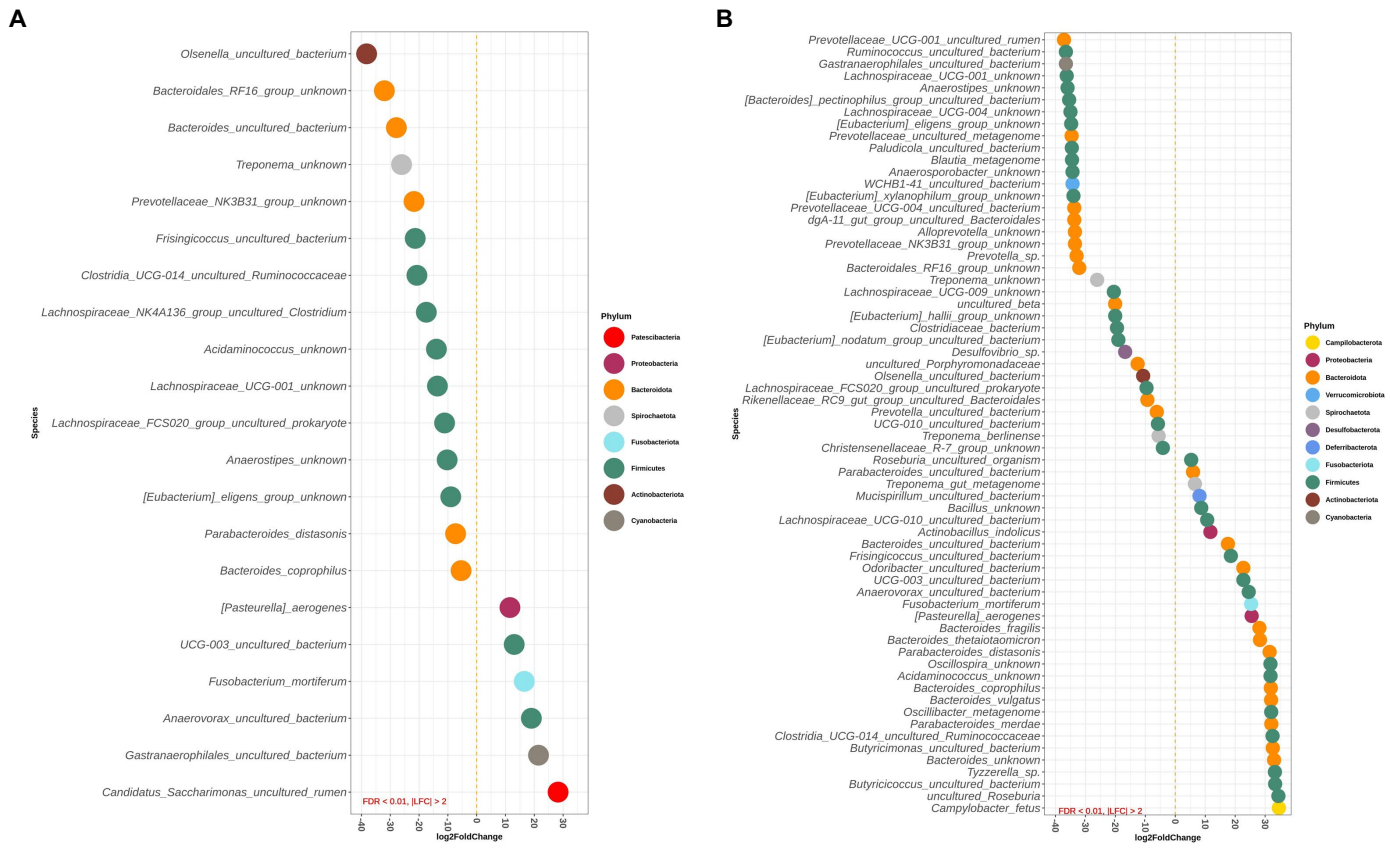
Waterfall plot for differentially abundant species of the main effect of diet (westernized diet groups vs. non-westernized diet groups; **A**) and DexSS (DexSS groups vs. non-DexSS groups; **B**). Different colors represent different phyla. Only species with |LFC| > 2 and FDR < 0.01 are presented.

Figures 4A,B depict differentially abundant species for the main effects of diet and DexSS administration with |LFC| > 2 and FDR < 0.01. Diet and DexSS administration impacted the abundance of 21 and 65 ASVs, respectively. The groups fed the westernized diet showed reduced abundance of 15 species, among which 8 species belonged to the Firmicutes phylum and 5 to the Bacteroidota, compared to those fed the standard diet. In addition, the westernized diet resulted in increased abundance of 6 species belonging to the Firmicutes and Patescibacteria phyla (Figure 4A). Administration of DexSS decreased the abundance of 35 species (Figure 4B), while it was associated with increased abundance of 30 species. Differentially abundant species associated with administration of DexSS were chiefly from the phyla Firmicutes and Bacteroidota followed by Spirochaetota, Desulfobacterota, and Proteobacteria.

Pairwise comparisons between treatment groups for species, where there was a significant interaction between the effect of diet and DexSS are presented in Supplementary Figure S2A for WD vs. CT, in Supplementary Figure S2B for DSS vs. CT, and in Supplementary Figure S2C for WD + DSS vs. CT. Compared to CT, the WD, DSS and WD + DSS groups had a different abundance of 25, 54, and 43 species, respectively. Animals in the WD group showed lower abundance of species, such as *Anaerovibrio* sp., *Lachnospiraceae* NK4A136_group uncultured organism, and *Olsenella umbonata*, and an increased abundance of *Helicobacter trogontum*, *Clostridium perfringens*, *Mogibacterium* sp., and *Roseburia hominis* compared with the CT group.

### 3.6. Concentration of short chain fatty acids and biogenic amines in colon and feces

Overall, except for valerate, which was constant in different sample types, the concentrations of all SCFAs varied according to the origin of the sample (Table 4). In proximal colon, the concentration of total SCFAs, acetate, propionate, and butyrate was higher than in distal colon but there was no difference between samples from the proximal colon and feces. On the other hand, valerate concentration was higher in fecal samples compared with those from the proximal and distal colon and concentration of iso-acids was constant across all sample types.

**Table 4:** Concentration (mmol/kg wet sample) of short-chain fatty acids (SCFA) in proximal and distal colon digesta and in fecal samples^1^.

	Sample type^2^		
	Proximal colon	Distal colon	Feces
SCFA	99.9 (88.0–114)^b^	82.7 (72.0–95.0)^a^	98.6 (85.0–115)^b^
Acetate	60.8 (53.0–70.0)^b^	48.9 (42.0–57.0)^a^	57.4 (49.0–68.0)^ab^
Propionate	22.0 (19.0–25.0)^b^	17.4 (15.0–20.0)^a^	20.1 (17.0–23.0)^b^
Butyrate	12.4 (10.0–15.0)^ab^	11.1 (9–14)^a^	13.2 (11.0–16.0)^b^
Valerate	2.60 (2.0–4.0)^a^	2.50 (2.0–3.0)^a^	3.30 (2.0–5.0)^b^
Iso-acids	1.70 (1.0–3.0)	2.10 (1.0–4.0)	3.30 (1.0–8.0)

1 Response values are reported with their corresponding 95% confidence intervals. Pairwise comparison of EMMs was adjusted using BH method and rows with different superscripts have different EMMs at *p* < 0.05.

2 Samples from different treatment groups are pooled in each segment, i.e., proximal colon (*n* = 23), distal colon (*n* = 22), and in feces (*n* = 21).

Table 5 and Supplementary Figure S3 show the concentration of SCFAs in the three sample types for the four treatment groups. These results were derived from the full model due to non-zero and relatively large coefficient for the second- and third-order interactions between treatment groups and sample type. Therefore, we report EMMs (with lower and upper confidence intervals) and pairwise comparison between groups. Although we did not observe statistical differences in the adjusted pairwise comparisons, possibly due to relatively high uncertainty in the estimates, there are numerical patterns in the effect of treatments on microbial metabolites that might have valuable biological meanings for future studies. In all sample types, WD and DSS groups showed numerically higher concentrations of SCFA and butyrate compared to CT, and WD + DSS the lowest compared to all other groups.

**Table 5:** Concentration (mmol/kg wet sample) of short-chain fatty acids (SCFA) measured in digesta from the proximal and distal colon and in feces.

	Groups^1^			
	CT	WD	DSS	WD + DSS
Proximal				
SCFA	96.4 (75.0–124)	110 (85.0–141)	114 (83.0–156)	82.8 (65.0–106)
Acetate	61.3 (47.0–80)	69.1 (53.0–90.0)	66.3 (47.0–93.0)	48.8 (37.0–64.0)
Propionate	20.9 (16.0–27.0)	22.8 (18.0–29.0)	25.2 (19.0–34.0)	19.4 (15.0–25.0)
Butyrate	10.1 (7.0–15.0)	12.5 (9.0–18.0)	17.1 (11.0–27.0)	10.8 (8.0–15.0)
Valerate	2.0 (1.0–4.0)	2.70 (1.0–5.0)	3.70 (2.0–8.0)	2.30 (1.0–4.0)
Iso-acids	1.70 (1.0–4.0)	2.40 (1.0–6.0)	1.50 (1.0–4.0)	1.40 (1.0–3.0)
Distal				
SCFA	74.3 (58.0–96.0)	91.2 (71.0–118)	95.4 (67.0–136)	72.5 (54.0–97.0)
Acetate	44.0 (34.0–58.0)	53.4 (41.0–70.0)	58.0 (39.0–86.0)	42.0 (30.0–58.0)
Propionate	15.4 (12.0–20.0)	17.4 (14.0–22.0)	20.2 (14.0–28.0)	17.1 (13.0–23.0)
Butyrate	10.1 (7.0–15.0)	12.8 (9.0–19.0)	13.5 (8.0–22.0)	8.80 (6.0–13.0)
Valerate	2.10 (1.0–4.0)	3.40 (2.0–6.0)	3.20 (1.0–7.0)	1.80 (1.0–3.0)
Iso-acids	2.50 (1.0–6.0)	3.90 (2.0–9.0)	1.30 (0.98–5.0)	1.30 (0.97–4.0)
Feces				
SCFA	88.3 (68.0–115)	120 (93.0–154)	94.2 (63.0–140)	94.9 (69.0–131)
Acetate	55.1 (41.0–73.0)	72.2 (55.0–94.0)	49.8 (32.0–78.0)	54.6 (38.0–79.0)
Propionate	17.7 (14.0–23.0)	22.6 (18.0–29.0)	20.5 (14.0–30.0)	20.0 (15.0–27.0)
Butyrate	9.30 (6.0–14.0)	14.2 (10.0–21.0)	17.1 (10.0–28.0)	13.4 (9–20)
Valerate	2.50 (1.0–5.0)	4.40 (2.0–8.0)	5.60 (3.0–12.0)	2.0 (1.0–4.0)
Iso-acids	3.40 (1.0–9.0)	6.0 (3.0–14.0)	3.40 (1.0–15.0)	1.60 (0.96–5.0)

Response EMM values are reported with their corresponding 95% confidence intervals.

1 Treatments group: control (CT; n = 17), westernized diet (CT + ground beef; *n* = 18), CT + dextran sodium sulphate (DSS; *n* = 9), WD + dextran sodium sulphate (WD + DSS; *n* = 13). Pairwise comparison for differences in EMMS between groups was adjusted with BH and EMMs are superscripted with different letters at *p.adjust* < 0.05.

Concentrations of biogenic amines in proximal and distal colon and feces are summarized in Table 6. In distal colon, the concentration of total biogenic amines was highest in the WD + DSS group (*p* < 0.05). In proximal and distal colon, concentration of putrescine was increased in the DSS and WD + DSS groups (*p* < 0.05) compared with CT; and in feces, it was highest in the WD + DSS group (*p* < 0.05). In general, concentrations of biogenic amines were highest in proximal and distal colon samples compared to feces (Supplementary Table S2).

**Table 6:** Concentration (mmol/kg wet sample) of biogenic amines in proximal colon, distal colon and fecal samples.

	Groups^1^			
	CT	WD	DSS	WD + DSS
Proximal				
Biogenic amines	162.5 (69.0–380)	356 (152–833)	280 (97.0–803)	535 (236–1,216)
Agmatine	16.7 (5.0–55.0)	18.8 (6.0–62.0)	31.3 (7.0–140)	46.3 (14.0–154)
Putrescine	38.0 (23.0–63.0)^a^	53.0 (32.0–87.0)^a^	143 (77.0–266)^b^	216 (132–352)^b^
Cadaverine	105 (36.0–310)	271 (93.0–792)	115 (30.0–440)	248 (87.0–706)
Distal				
Biogenic amines	135 (58.0–313)^a^	187 (80.0–438)^ab^	371 (120–1,142)^ab^	564 (226–1,403)^b^
Agmatine	14.2 (4.0–47.0)	15.1 (5.0–50.0)	35.7 (6.0–199)	65.0 (15.0–281)
Putrescine	34.1 (21.0–56.0)^a^	39.0 (24.0–64.0)^a^	213 (106–427)^b^	232 (130–415)^b^
Cadaverine	81.1 (28.0–236)	116 (40–340)	121 (29.0–500)	253 (80.0–799)
Feces				
Biogenic amines	177.2 (73.0–428)	144 (61–338)	215 (62.0–744)	389 (141–1,069)
Agmatine	12.6 (3.0–47.0)	12.2 (4.0–40.0)	17.9 (2.0–151)	23.7 (4.0–141)
Putrescine	30.4 (18.0–52.0)^a^	25.3 (15.0–42.0)^a^	43.7 (19.0–99)^a^	168 (84.0–336)^b^
Cadaverine	107 (35.0–328)	91.6 (31.0–269)	132 (28.0–625)	202 (58.0–707)

1 Treatments group: control (CT; *n* = 17), westernized diet (CT + ground beef; *n* = 18), CT + dextran sodium sulphate (DSS; *n* = 9), WD + dextran sodium sulphate (WD + DSS; *n* = 13). Pairwise comparison for differences in EMMS between groups was adjusted with BH and EMMs are superscripted with different letters at *p.adjust* < 0.05.

**Figure 5:**
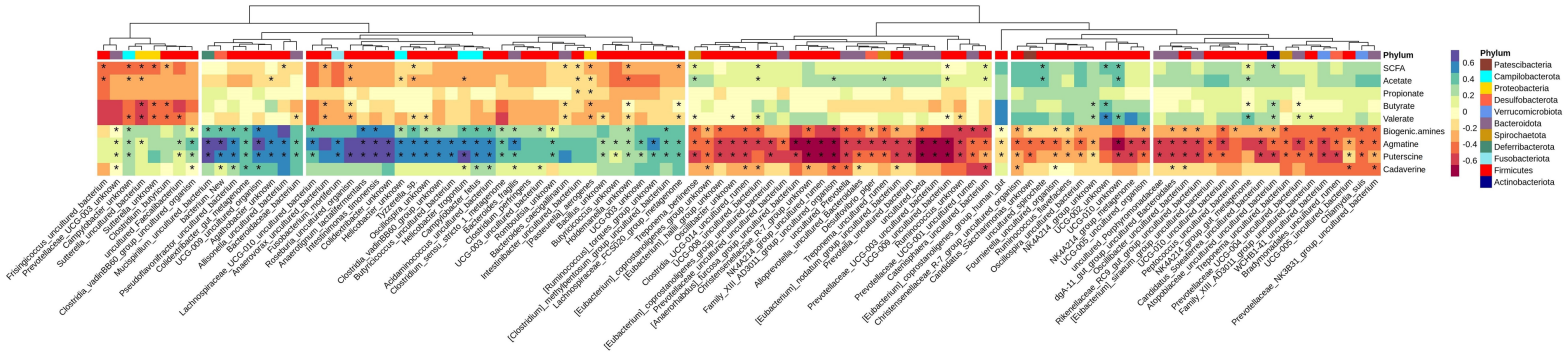
Heatmap of Spearman’s rank correlation coefficient (*r_s_*) between the top 100 abundant species and SCFA and biogenic amines in all segments. Columns are clustered based on the standard deviations between species abundances, i.e., those with lower deviation are clustered together. Species are color-labeled with their correspondent phyla and significant associations are labeled with stars.

Variations between colonic segments was inconsiderable for beta diversity and differentially-abundant taxa specifically; therefore, the microbiota data were pooled for samples taken from the different segments to evaluate the association of top 100 abundant species (chosen based on the highest standard deviation in the association matrix) with fermentation products by a Spearman rank test. Figure 5 shows that concentration of SCFAs in the large intestine was positively associated with 9 species and negatively associated with 14 species (*p* < 0.05), mainly from Firmicutes. Five species (mainly from Bacteroidota, Actinobacteriota, and Firmicutes) were positively associated with butyrate production, while 9 species (mainly belonged to Firmicutes and Proteobacteria) had a negative association with butyrate concentration. Total concentration of biogenic amines showed a positive association with 25 species and negative association with 34 species (*p* < 0.05).

## 4. Discussion

Due to technical variations in 16 s rRNA gene amplicons as the result of differences in the quality of sequencing machines and individual runs, the data generated from next generation sequencing (NGS) machines are often treated as relative abundance rather than their absolute counts (Fernandes et al., 2014). However, since reads in the compositional data have an inherit mutual dependence, i.e., with increase in one feature, the other should decrease to maintain the overall sum, relative abundant NGS data can lead to misinterpretation of microbial community structure (Jian et al., 2020). This mutual dependence, therefore, makes it difficult to build a reliable biological conclusion over the mere effect of the environmental factor on the microbial phylotypes (Galazzo et al., 2020); it also hampers the actual detection of the direction and the extent of changes in taxa abundance (Morton et al., 2017; Props et al., 2017). For quantitative microbiome profiling in their absolute counts, different techniques such as flow cytometry (for total cell count; Props et al., 2017), and DNA-based methods like qPCR (Liu et al., 2015) and spike-in bacteria (Stämmler et al., 2016) have been suggested to normalize the relative abundance data. In the present study, the results of absolute (from qPCR 16 s rRNA copy-number genes) abundance of NGS data are presented.

Despite efforts to better understand the pathology of IBD, the underlying mechanisms associated with these diseases are not yet understood. Disruption in the compositional balance of gut microbiota, also known as dysbiosis, resulting from dietary factors, medication, oxygen availability, or immune reactions, has been associated with IBD (Wei et al., 2021). Among dietary factors, red meat, as an important constituent of a typical western diet, has repeatedly been identified as a risk factor for the incidence of IBD due to dysbiosis of gut microbiota (Le Leu et al., 2013; Li et al., 2021). Nielsen et al. (2020) showed that administering DexSS induced colitis in pigs and that performance (e.g., feed intake and average daily gain) was negatively affected by treatment with DexSS. Furthermore, the inflammation scores in the colon and colonic expression of pro-inflammatory cytokines such as interleukin 6 (IL-6) were increased when providing DexSS (Nielsen et al., 2020). This is in agreement with previous studies, reporting that DexSS administration successfully induced UC in porcine (Pistol et al., 2020) and murine (Eichele and Kharbanda, 2017) models. In our study, DexSS administration was associated with a significant reduction in the total number of ASVs and alpha diversity in colonic and fecal samples. Previous studies have also reported reduced diversity of gut microbiota in patients with IBD (Ott et al., 2004; Scanlan et al., 2006; Schirmer et al., 2019). According to Nielsen et al. (2020), although feeding a westernized diet by adding beef did not result in inflammation and expression of pro-inflammatory cytokines, it exacerbated the clinical and histological signs of colitis in pigs challenged with DexSS. In the current study, the same westernized diet did not affect alpha diversity in pigs, while it significantly resulted in differential abundance of phyla (Figure 3A) and species compared to a control group (Supplementary Figure S2A). Nevertheless, the impact of the westernized diet on beta diversity was relatively small and, in general, gut microbiota in the WD group had uniquely more species in common with the CT group (n = 54) than with DSS and WD + DSS animals, indicating that adding 15% beef to a control pig diet, with higher fiber content than a typical human western diet, as a model for a westernized diet may be insufficient to provoke the microbial shifts that lead to dysbiosis-related disease. On the other hand, the experimental period in the current study might have been too short in order to see a more significant impact on gut microbial composition or dysbiosis.

### 4.1. Bacterial composition

Most species affected by diet and DexSS administration belonged to Firmicutes, Bacteroidota, Actinobacteriota and Proteobacteria, which represent core phyla in human (Alam et al., 2020) and pig (Holman et al., 2017) gut microbiota. Both, feeding the westernized diet and administering DexSS resulted in an increased abundance of Fusobacteriota, a result which has previously been reported in piglets with diarrhea (Hermann-Bank et al., 2015) and in patients with UC (Rajilić-Stojanović et al., 2013). *Fusobacterium mortiferum*, belonging to the Fusobacteriota, was increased as a result of feeding the westernized diet and of administrating DexSS (LFC > 10), and *Fusobacterium* sp. has been characterized as an opportunistic pathogen that thrives in patients with UC (Rajilić-Stojanović et al., 2013). Nevertheless, the impact of *F. mortiferum* on UC development is difficult to discern since it has been reported that a strain of *F. mortiferum* inhibited the growth of both Gram-negative and Gram-positive bacteria by producing a bacteriocin-like substance (Portrait et al., 2000). In the current study, the abundance of Actinobacteriota was slightly decreased in the groups receiving the westernized diet and in those receiving DexSS as compared to those fed the standard diet and those not receiving DexSS, respectively. This corresponded to the reduced abundance of an uncultured *Olsenella* species belonging to this phylum. Pairwise comparison between treatments also showed lower abundance of *O. umbonata* in the WD group than in the CT group (Supplementary Figure S2A). Compared to the CT group, the DSS group had lower abundance of *Bifidobacterium pseudolongum* and higher of *O. umbonata* and of an uncultured *Colinsella* species. *Olsenella umbonata* and *Colinsella* are Gram-positive lactic-acid-producing bacteria belonging to Actinobacteriota phylum (Kraatz et al., 2011), which *O. umbonata* might be involved in regulating the expression of SCFA receptors in the gut epithelium (Wang et al., 2020). Other studies have reported increased abundance of the Actinobacteriota phylum in patients with UC (Alam et al., 2020; Putignani et al., 2021), while Alam et al. (2020) reported a reduction of Actinobacteriota at class (*Coriobacteriia*) and family (*Bifidobacteriaceae*) levels compared to healthy groups. Therefore, to get a better picture of bacterial association with the disease status, studying changes at ASV/species taxonomic level would be more informative over changes at phylum level.

At ASV level, in animals fed westernized diet, we observed significantly reduced abundance of species from Firmicutes, Bacteroidota and Actinobacteriota phyla, which are mainly involved in carbohydrate fermentation such as *Lachnospiraceae* NK4A136_group, an uncultured organism at species level within Firmicutes. *Lachnospiraceae* NK4A136_group was also observed at lower abundances in DexSS-treated pigs, which is in agreement with previous studies reporting reductions of these ASVs in mice challenged with DexSS and fed a diet high in red meat (Li et al., 2021). Ma et al. (2020) reported that the *Lachnospiraceae* NK4A136 group are SCFA-producing bacteria and its increased abundance was correlated with enhanced gut barrier function in mice. In contrast, our results could not confirm a positive correlation between *Lachnospiraceae* NK4A136_group and the concentration of SCFA. However, the DSS group had lower abundance of *Lachnospiraceae* NK4A136_group, when compared to CT group. *Bacteroides vulgatus*, a pathogenic *Bacteroides* in the development of IBD (Shiba et al., 2003), was increased in pigs challenged with DexSS. This was in agreement with the results of Galipeau et al. (2021), who observed increased abundance of *B. vulgatus*, expressing proteolytic activity. Moreover, the abundances of other pathogens like *B. coprophilus* (Bacteroidota phylum), *Clostridium perfringens* (Firmicutes phylum), *H. trogontum* and *Campylobacter fetus* (both Campilobacterota phylum), which have been reportedly associated with IBD in humans (Loss et al., 1980; Hansen et al., 2011; Banaszkiewicz et al., 2014; Wu et al., 2021), were increased in DexSS-treated animals. The WD group also resulted in increased abundance of *C. perfringens* and *H. trogontum* compared with CT group*. Clostridium perfringens* seems to be the most well-documented pathogen linked to IBD (Banaszkiewicz et al., 2014), which produces an enterotoxin that causes damages to the villus tip cells by binding to their receptors and forming pores (Smedley III et al., 2008) that may disrupt the integrity of colonic epithelium. Nomura et al. (2021) reported that *Parabacteroides merdae* (Bacteroidota phylum) was among other Bacteroidota species showing a strong correlation with the UC activity metrics and we observed a significant increase in the abundance of this species in DexSS-treated pigs. In agreement with our results, Galipeau et al. (2021) also reported a significant increase in the relative abundance of *P. merdae* in the feces of individuals prior to the onset of UC; and in those with active UC, its abundance was significantly higher than in healthy, control groups. This can suggest this species as a potential biomarker for monitoring UC.

### 4.2. Impact of the treatments on microbial metabolites

Short-chain fatty acids, in particular butyrate, improve gut maturity and health, and help maintain gut integrity and barrier functionality (Panah et al., 2021). In different studies, the reduced diversity of carbohydrate-degrading anaerobic species in the colon such as *Lachnospiraceae* and *Ruminococcaceae* families (Schirmer et al., 2019) and Bacteroides, Eubacterium, and *Lactobacillus* species (Ott et al., 2004) have been associated with the development of IBD. In the present study, WD-fed animals exhibited an increase in the abundance of *Pasteurella aerogenes* and *Anaerovorax uncultured bacterium*, which were negatively correlated with the concentration of SCFA (Figure 5). On the other hand, WD-fed animals showed a decreased abundance of an uncultured *Frisingicoccus* species and the genus *Sutterella*, which also had a negative association with SCFA concentration. In DexSS-treated groups, the abundance of an uncultured *Anaerovorax* and *Roseburia uncultured organism* increased, and the abundances of both were negatively correlated with the production of SCFAs. However, the effects of WD and DSS on SCFAs did not completely follow the changes in the abundance of species correlated with SCFA production. Due to the presence of uncertainties in our estimated EMMs and perhaps also due to rather small sample sizes, we could not capture statistically significant differences in SCFA concentrations, while their numerical variations due to treatment effects might be of interest for future studies. In colon and in fecal samples, WD and DSS groups had numerically higher concentration of SCFAs regardless of the fact that in these animals, species negatively correlating with SCFA production, were in higher abundance. Butyrate was also numerically higher in the DSS and WD groups compared to CT animals in both colonic digesta and fecal samples, while many studies have reported an alleviating effect of butyrate on IBD severity (Chen et al., 2018; Silva et al., 2018; Couto et al., 2020). An explanation to this may in part be due to alterations in the capacity of the colonic epithelium to transport and oxidize butyrate, caused by feeding the westernized diet and DexSS administration. As shown in Table 4, overall, from proximal to distal colon, 22.9% of total produced SCFAs concentration was reduced, while that for WD, DSS, and WD + DSS groups was 16.3, 16.3, and 12.4%, respectively. Ferrer-Picón et al. (2020) also reported that reduced butyrate-producing bacteria in the feces of active IBD patients did not correlate with the concentration of butyrate recovered from fecal samples. They related this phenomenon to the fact that increased pro-inflammatory cytokine TNF-α in the inflamed colon of IBD patients rendered the epithelium less responsive to butyrate. Likewise, De Preter et al. (2011) reported that butyrate oxidation in the colonic epithelium of patients with UC was lower than in healthy controls and increasing the concentration of butyrate did not result in a higher oxidation of butyrate in the epithelium. Although this needs to be further investigated, it could be speculated that WD and DexSS rendered colonic epithelium less responsive to butyrate oxidation. On the other hand, Nielsen et al. (2020) did not observe any significant changes in the expression of mRNA related to TNF-α in the colonic mucosa of pigs receiving the westernized diet and/or DexSS. Furthermore, our results showed that except for valerate, the concentration of total SCFA and individual SCFAs in proximal colon and in feces were not different as it decreased from proximal through distal colon and increased again in feces. This might indicate that fecal samples could represent the concentration of SCFAs formed in proximal colon, but it may not be a good representative sample for explaining the fate of SCFAs across the colon, i.e., from proximal to distal colon.

Biogenic amines are produced by gut bacteria through decarboxylation of amino acids and their recovery in feces was reported to be a relevant biomarker for IBD (Maráková et al., 2020). The concentration of putrescine increased by administering DexSS and the effect was exacerbated when combined with the westernized diet, with a similar trend observed for total biogenic amine concentration. This was corresponded with increased abundance of species positively correlated with biogenic amine concentration. Changes in microbiota composition and/or impaired absorption capacity of the inflamed colon epithelium could be contributing factors to the observed higher concentration of biogenic amines in the DSS group. An increased substrate availability, i.e., more protein, by providing red meat to the DSS-challenged animals (WD + DSS) and a more severe inflammation seen in this group (Nielsen et al., 2020) would then lead to the exacerbated values measured in this group. Gobert et al. (2018) and Liu et al. (2020) also observed increased concentration of putrescine in DexSS-treated mice with a positive correlation to the inflammation scale of challenged mice.

The exact mechanisms behind our observations remain unclear and further investigations on the epithelial oxidative capacity in relation to SCFAs, especially butyrate, and functional analysis of gut microbiota in patients with UC are required. In agreement with the findings of Nielsen et al. (2020), who suggested potential worsening effects of westernized diet on UC status, westernized diet could be a risk factor and an exacerbating agent for IBD by reducing SCFA-producing bacteria, increasing the abundance of pathogens and microbial proteolytic activity, and possibly by reducing butyrate absorption and oxidation capacity of colonic epithelium. Nevertheless, these results were driven from a relatively small sample size of animals (n = 5 or 6) per treatment and require caution in interpretation as also stated by Nielsen et al. (2020).

The current study was conducted with pigs; therefore, a typical pig diet was used as a basis to add the beef meat. Pig diets have a relatively higher fiber content than a typical human diet in the Western countries. This fact has probably affected the results obtained regarding the impact of adding beef to the diet, as indicated by Pituch-Zdanowska et al. (2015), supplementation of some types of dietary fiber can help alleviate lesions of the intestinal mucosa during the course of the UC disease.

## 5. Conclusion

We observed that the westernized diet, especially in combination with DexSS, resulted in significant changes in abundance of bacterial species involved in SCFA production. Providing DexSS lead to increased biogenic amine concentration, mainly putrescine in colon and in feces, and the effect was exacerbated when the westernized diet was fed to DexSS-challenged pigs. However, further investigations are required for studying the mechanisms behind the changes observed. We also confirm a significant change in colonic and fecal microbial composition in DexSS-induced UC pigs. Using a pig model in which the control diet has a lower fiber content, simulating more a human diet, would most probably be a better model to be used as surrogate for humans when investigating UC in relation to dietary interventions.

## Data availability statement

The datasets presented in this study can be found in online repositories. The names of the repository/repositories and accession number (s) can be found at: https://www.ncbi.nlm.nih.gov/, PRJNA867563.

## Ethics statement

The animal study was reviewed and approved by The Danish Ministry of Justice, Animal Testing Act no. 1306 of 23 November 2007.

## Author contributions

FP was responsible for bioinformatics and statistical analysis and for writing the first draft of the manuscript. KN performed all DNA-based analyses under the guidance of AndS and the microbial metabolite analysis under the guidance of NC. GS contributed to checking statistical and bioinformatics analysis of the data and correcting the manuscript for language and grammar. AnnS contributed to checking bioinformatics analysis and contributed to scientific proof reading of the manuscript. AndS, CL, TN, OH, and SP contributed to scientific proof reading of the manuscript. MF conducted the animal experimentation. NC contributed to scientific proof reading of the manuscript and she is the study responsible. All authors contributed to the article and approved the submitted version.

## Funding

This research was supported by the Independent Research Fund Denmark, grant no. 1335-00116.

## Conflict of interest

The authors declare that the research was conducted in the absence of any commercial or financial relationships that could be construed as a potential conflict of interest.

## Publisher’s note

All claims expressed in this article are solely those of the authors and do not necessarily represent those of their affiliated organizations, or those of the publisher, the editors and the reviewers. Any product that may be evaluated in this article, or claim that may be made by its manufacturer, is not guaranteed or endorsed by the publisher.
