# Corrigendum: A westernized diet changed the colonic bacterial composition and metabolite concentration in a dextran sulfate sodium pig model for ulcerative colitis

**DOI:** 10.3389/fmicb.2023.1238157

**Published:** 2023-06-27

**Authors:** Farhad M. Panah, Katrine D. Nielsen, Gavin L. Simpson, Anna Schönherz, Andreas Schramm, Charlotte Lauridsen, Tina S. Nielsen, Ole Højberg, Marlene Fredborg, Stig Purup, Nuria Canibe

**Affiliations:** ^1^Department of Veterinary and Animal Sciences, Aarhus University, Tjele, Denmark; ^2^Department of Biology, Aarhus University, Aarhus, Denmark

**Keywords:** inflammatory bowel disease, ulcerative colitis, meat consumption, colonic inflammation, porcine model, 16S rRNA gut metagenomics, dextran sulfate sodium

In the published article, there was an error in the **Legend** for Tables 2 and 3. The Table legends should be corrected as follows:

**Table 2:** Alpha diversity metrics with their estimated marginal means and their 95% confidence interval for each treatment group.

	**Groups** ^**1**^			
	**CT**	**WD**	**DSS**	**WD** + **DSS**
**Proximal**				
Chao1	533 (426–665)^b^	500 (399–625)^ab^	460 (360–588)^ab^	367 (296–462)^a^
Shannon	5.30 (5.0–5.66)^b^	5.20 (4.92–5.57)^b^	5.0 (4.63–5.30)^ab^	4.80 (4.05–5.09)^a^
Faith PD	35.4 (30.0–42.0)^b^	35.5 (30.0–42.0)^b^	29.4 (24.0–35.0)^ab^	27.4 (23.0–32.0)^a^
**Distal**				
Chao1	535 (428–668)^b^	555 (443–695)^b^	397 (311–508)^a^	397 (315–501)^a^
Shannon	5.30 (4.94–5.59)^b^	5.30 (4.96–5.63)^b^	4.80 (4.46–5.12)^a^	4.90 (4.59–5.21)^ab^
Faith PD	36.4 (31.0–43.0)^b^	38.6 (32.0–46.0)^b^	26.9 (22.0–32.0)^a^	28.5 (24.0–34.0)^a^
**Feces**				
Chao1	488 (387–615)	534 (426–668)	391 (301–506)	434 (347–542)
Shannon	5.20 (4.90–5.57)	5.30 (4.95–5.62)	5.0 (4.64–5.35)	5.0 (4.71–5.33)
Faith PD	34.3 (29–41)^ab^	37.9 (32–45)^b^	27.7 (23–34)^a^	30.6 (26–36)^ab^

^1^Treatment groups: control (CT; *n* = 17), WD (CT + ground beef; *n* = 18), CT + dextran sulfate sodium (DSS; *n* = 14), WD + dextran sulfate sodium (WD + DSS; *n* = 17). Pairwise comparison for differences in EMMS between groups was adjusted with BH and EMMs are superscripted with different letters at *p* < 0.05.

**Table 3:** Test statistics of the dbRDA model for the effect of treatment and sample type on Bray–Curtis dissimilarity.

	**Degree of freedom**	**% of variance explained**	**Sum of squares**	**Pseudo-F**	***P-*value**
WD	1.0	5.0	0.05	4.90	0.02
DSS	1.0	22.0	0.23	21.1	< 0.01
WD **·** DSS	3.0	31.0	0.33	10.8	< 0.01
Sample type	2.0	1.0	0.12	0.55	< 0.01

The values are averaged over three blocks.

The authors apologize for this error and state that this does not change the scientific conclusions of the article in any way. The original article has been updated.

