# Supplementary material for: A westernized diet changed the colonic bacterial composition and metabolite concentration in a dextran sulfate sodium pig model for ulcerative colitis
Source: Front Microbiol. 2023 Apr 17;14:1018242. doi: 10.3389/fmicb.2023.1018242 (PMC10150118; doi:10.3389/fmicb.2023.1018242)
Supplement: Supplementary file 1 [file Data_Sheet_1.docx]

Supplementary Material

# Supplementary Figures and Tables

## Supplementary Tables

**Table S1:** Alpha diversity metrics with their estimated marginal means and their 95% confidence interval for each segment. The values are the average of samples from the four treatment groups in the three blocks.

|  | Sample type^1^ | |  |
| --- | --- | --- | --- |
| Alpha indices | Proximal colon | Distal colon | Feces |
| Chao1 | 461 (413-515) | 465 (416-520) | 458 (409-513) |
| Shannon | 5.1 (4.92-5.23) | 5.0 (4.89-5.20) | 5.1 (4.97-5.29) |
| Faith PD | 31.7 (29-35) | 32.2 (30-35) | 32.4 (30-35) |

^1^Samples from different treatment groups are pooled in each segment, i.e. proximal colon (n = 23), distal colon (n = 22) and in feces (n = 21). Pairwise comparison for differences in EMMS between groups was adjusted with BH and EMMs are superscripted with different letters at *P.adjust* < 0.05.

**Table S2.** Concentrations (mmol/kg wet sample) of biogenic amines in separate segments; proximal and distal colon digesta and in fecal samples^1^.

|  | Sample type^2^ | | |
| --- | --- | --- | --- |
|  | Proximal colon | Distal colon | Feces |
| Biogenic amines | 305 (198-469) | 269 (171-423) | 215 (132-349) |
| Agmatine | 25.9 (14.0-46.0) | 26.7 (14.0-51.0) | 15.9 (8.0-33.0) |
| Putrescine | 88.9 (69.0-114) ^b^ | 90.1 (68.0-119) ^b^ | 48.8 (36.0-67.0) ^a^ |
| Cadaverine | 169 (98-291) | 130 (74.0-231) | 127 (69.0-234) |

^1^Tyramine and DL-methionine were below detection level. concentration was below detection level (3.2 mg/kg wet sample). Values are reported with their corresponding EMMs and their 95% confidence intervals.
^2^Samples from different treatment groups are pooled in each segment, i.e. proximal colon (n = 23), distal colon (n = 22) and in feces (n = 21). Pairwise comparison for differences in EMMS between groups was adjusted with BH and EMMs are superscripted with different letters at *P.adjust* < 0.05.

## Supplementary Figures


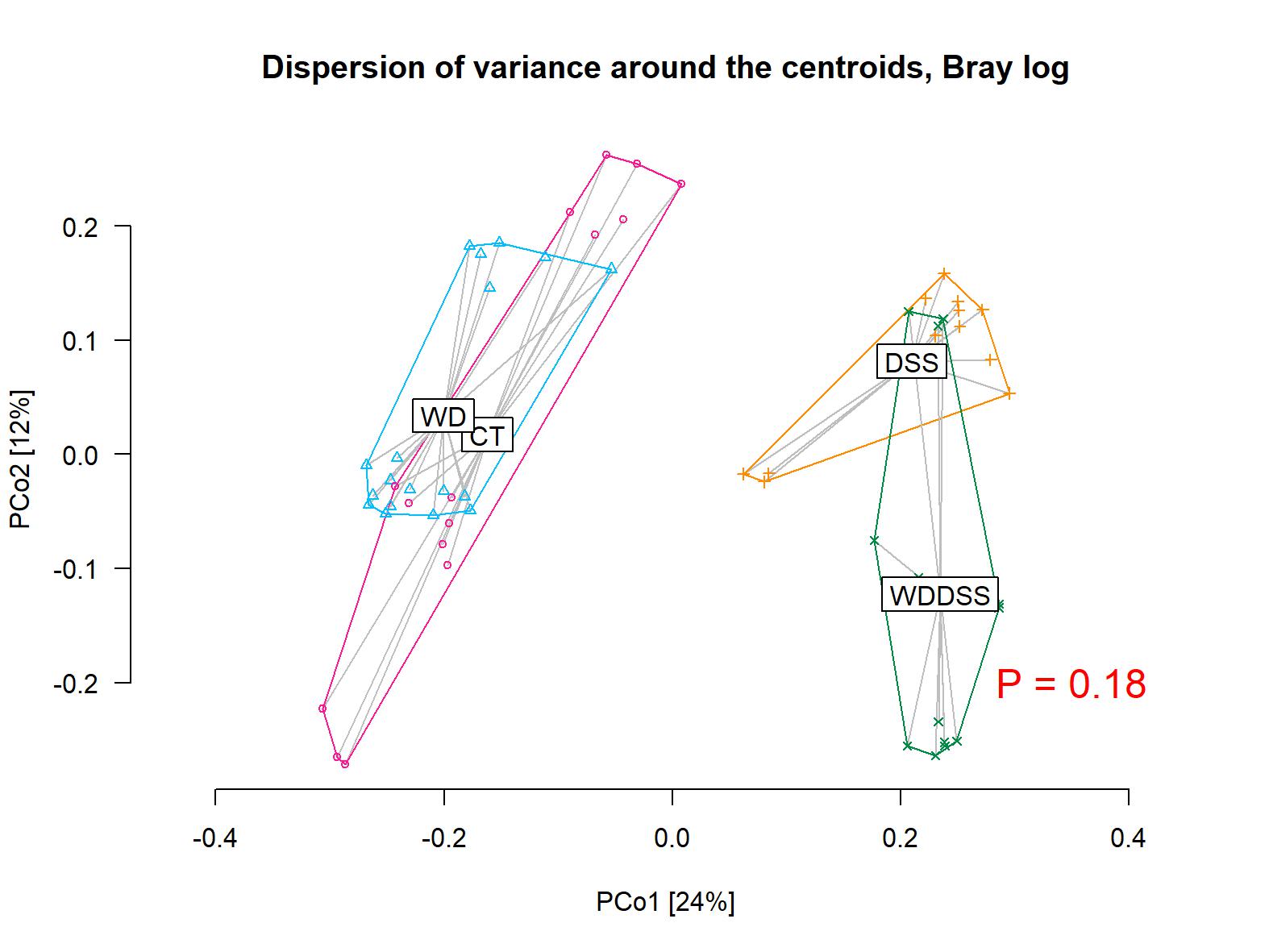


**Figure S1.** PCoA ordination plot of variance homogeneity around the centroids in treatments (CT: control, WD: westernized diet, DSS: dextran sodium sulfate, and WDDSS: westernized diet+DSS) based on Weighted UniFrac phylogenetic distance (WUFPD; *F*-statistics = 2.23). The *P-*value of > 0.06 indicates some evidence of heterogeneity for variance in each treatment group.


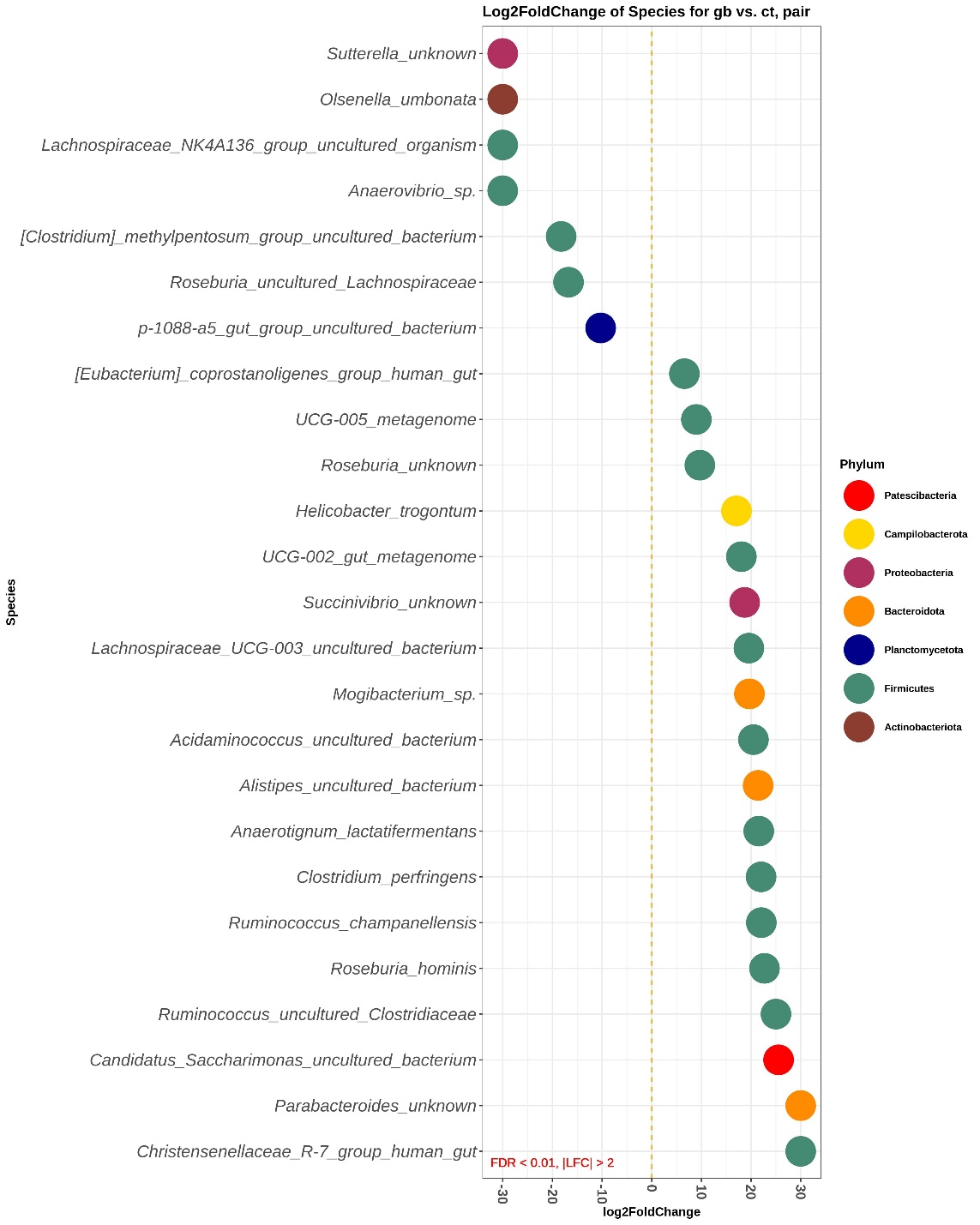


**A**


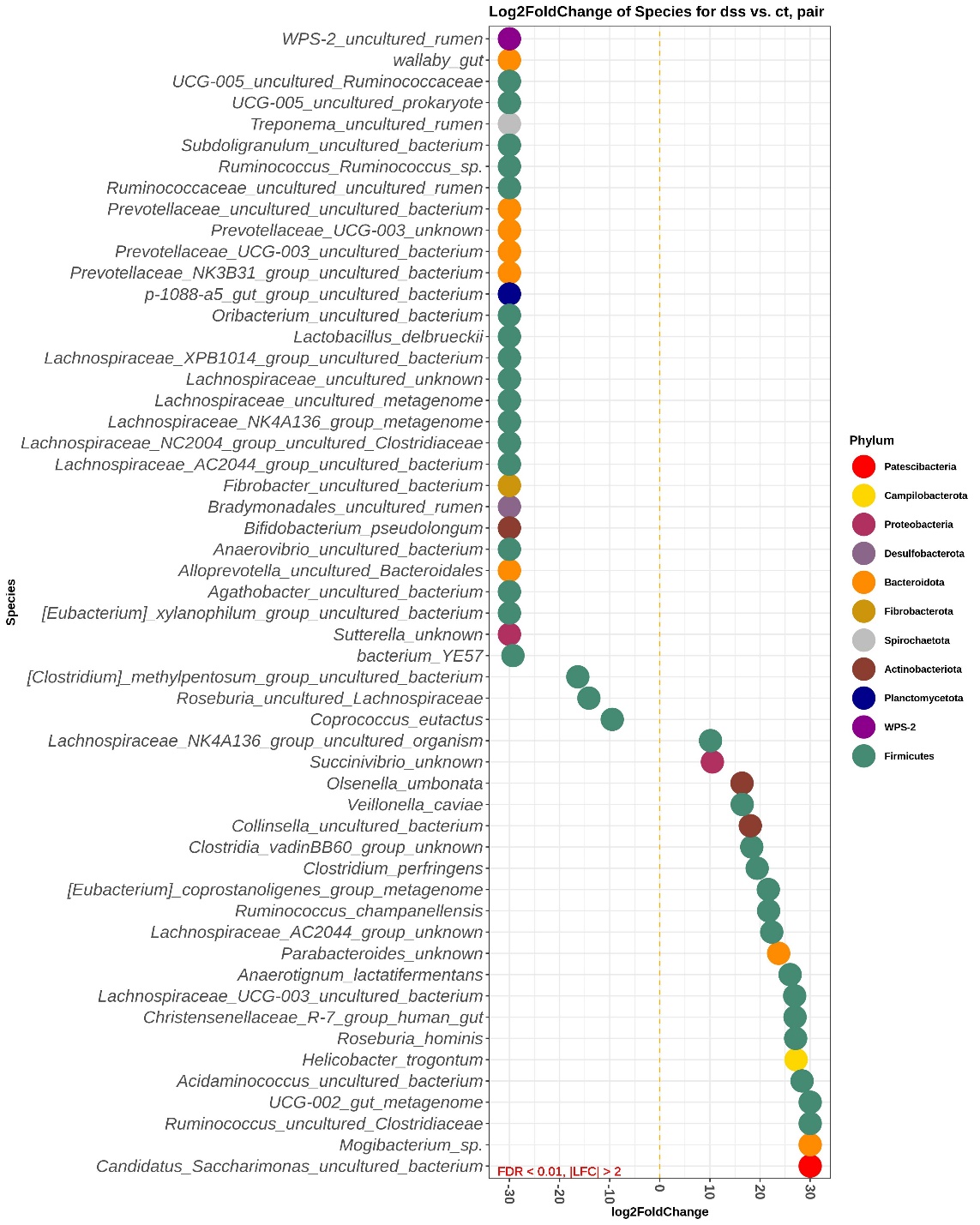


**B**


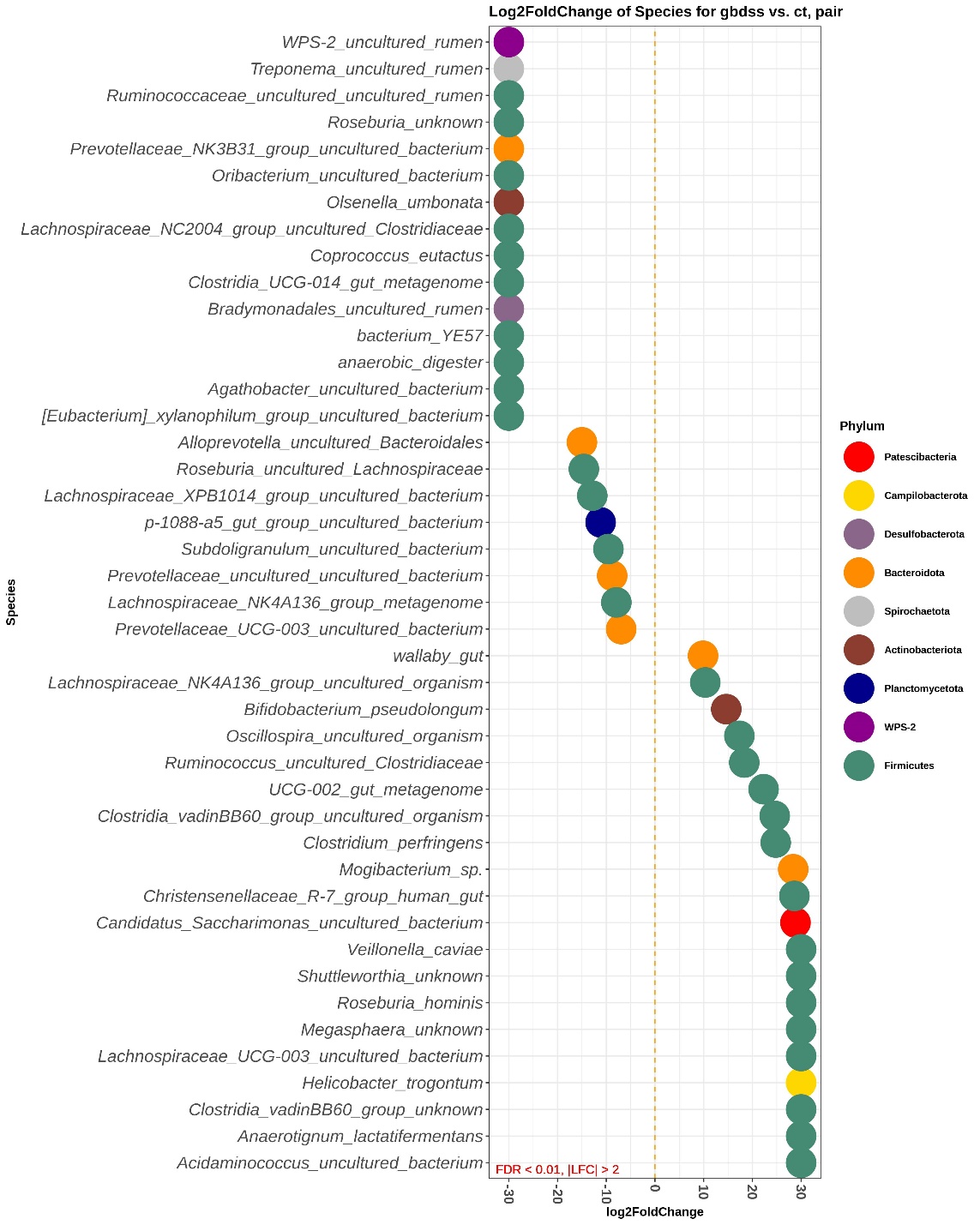


**C**

**Figure S2.** Pairwise comparison of species with significant interaction for WD vs. CT (**A**), DSS vs. CT (**B**) and WD+DSS vs CT (**C**). Different colors represent different phyla. Only species with |LFC| > 2 and FDR < 0.01 are presented.


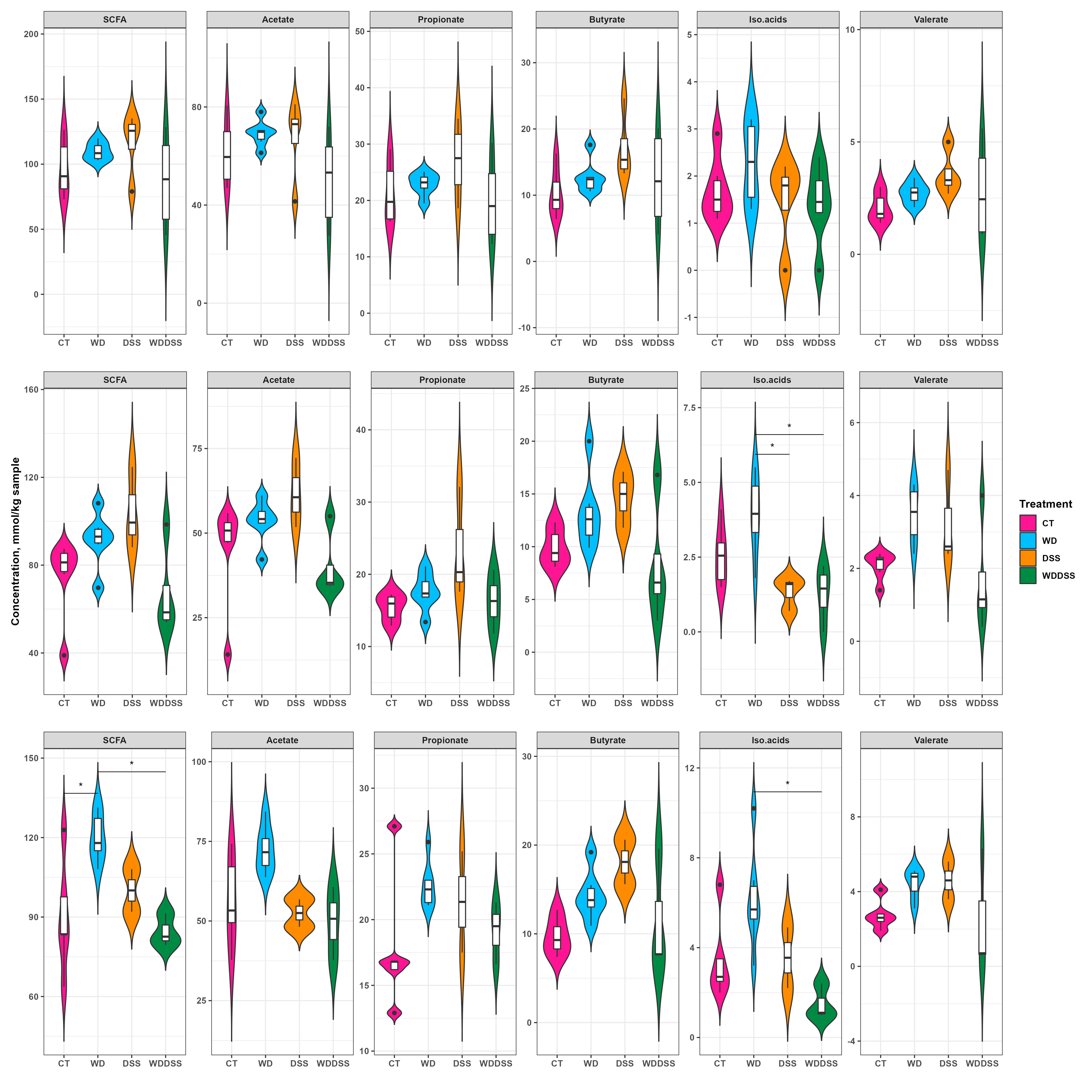


**A**

**B**

**C**

**Figure S3.** Concentration (mmol/kg wet sample) of total and individual short-chain fatty acids (SCFA) in proximal colon (A), distal colon (B) and in feces (C). Iso-acids are the sum of iso-butyrate and iso-valerate. Means are compared with an emmeans test and *p*-values were adjusted with BH method.
